# Storage of Titanium Dental Implants in Ozone Nanobubble Water Retards Biological Aging and Enhances Osseointegration: An In Vivo Study

**DOI:** 10.3390/ma18133156

**Published:** 2025-07-03

**Authors:** Hidehiro Horikawa, Tomoo Yui, Yasuhiro Nakanishi, Yukito Hirose, Takashi Kado, Takashi Nezu, Hourei Oh, Morio Ochi

**Affiliations:** 1 Division of Fixed Prosthodontics and Oral Implantology, Department of Oral Rehabilitation, School of Dentistry, Health Sciences University of Hokkaido, Tobetsu 061-0293, Japan; niyas.kor.kamuy@gmail.com (H.H.); nakanisi@hoku-iryo-u.ac.jp (Y.N.); yukito@hoku-iryo-u.ac.jp (Y.H.); 2 Division of Clinical Cariology and Endodontology, Department of Oral Rehabilitation, School of Dentistry, Health Sciences University of Hokkaido, Tobetsu 061-0293, Japan; yuit@hoku-iryo-u.ac.jp; 3 Division of Periodontology and Endodontology, Department of Oral Rehabilitation, School of Dentistry, Health Sciences University of Hokkaido, Tobetsu 061-0293, Japan; kado@hoku-iryo-u.ac.jp; 4 Division of Biomaterials and Bioengineering, School of Dentistry, Health Sciences University of Hokkaido, Tobetsu 061-0293, Japan; tnezu@hoku-iryo-u.ac.jp; 5 The Center of Innovation in Dental Education, Osaka Dental University, 8-1, Kuzuhahanazono-cho, Hirakata-shi 573-1121, Japan; ohoh@cc.osaka-dent.ac.jp

**Keywords:** dental implants, titanium, osteointegration, ozone, nanobubbles

## Abstract

The biological aging of titanium implants, marked by increased surface hydrophobicity and organic contamination, reduces bioactivity and delays osseointegration. A major challenge in implant dentistry is determining how to preserve surface hydrophilicity during storage, as conventional atmospheric conditions accelerate surface degradation. This pilot in vivo study aimed to evaluate ozone nanobubble water (NBW3) as a storage medium to prevent biological aging and enhance the early-stage osseointegration of glow discharge-treated titanium implants. Screw-type implants were stored in either NBW3 or atmospheric conditions and then implanted into femoral bone defects in Sprague Dawley rats. Removal torque testing, scanning electron microscopy (SEM), energy-dispersive X-ray spectroscopy (EDX), and histological analysis of bone-to-implant contact (BIC) were performed 14 and 28 days post-implantation. At 14 days, the NBW3-stored implants demonstrated significantly higher removal torque (2.08 ± 0.12 vs. 1.37 ± 0.20 N·cm), BIC (65.74 ± 12.65% vs. 44.04 ± 14.25%), and Ca/P atomic ratio (1.20 ± 0.32 vs. 1.00 ± 0.22) than the controls. These differences were not observed at 28 days, indicating NBW3’s primary role in accelerating early osseointegration. The findings suggest that using NBW3 is a simple, effective approach to maintain implant surface bioactivity during storage, potentially improving clinical outcomes under early or immediate loading protocols.

## 1. Introduction

Although dental implants generally exhibit high success rates, early failure—defined as failure occurring before prosthetic loading—remains a significant clinical concern. Epidemiological studies typically report early failure rates ranging from 3% to 5%, with various contributing factors. For instance, an early failure rate of 4.0% has been associated with severe periodontitis and the use of short implants [1]. Similarly, a 3.8% failure rate has been linked to an inadequate keratinized mucosa width and a reduced implant diameter [2]. Smoking and implant placement in the maxillary molar region have also been correlated with a 4.0% early failure rate [3].

The long-term clinical success of dental implants is critically dependent on achieving direct bone apposition onto the titanium surface without the interposition of soft or connective tissue. Brånemark et al. first reported the potential for osseointegration between bone and titanium [4]. Subsequent studies have demonstrated that the morphological and physicochemical characteristics of titanium surfaces play pivotal roles in promoting osseointegration [5,6,7,8,9,10]. Titanium, widely regarded as the material of choice for dental implants, is classified as biologically inert and exhibits excellent biocompatibility [11]. However, titanium surfaces undergo biological aging almost immediately after processing [12], primarily due to surface oxidation. This oxidation disrupts the hydrogen bonding network of water molecules on the titanium oxide layer and alters surface wettability [13,14]. Furthermore, organic contamination intensifies these effects: while oxygen-containing functional groups such as hydroxyl groups support hydrogen bonding with water, organic impurities mask intrinsic surface properties and contribute to increased hydrophobicity [15]. Consequently, surface aging remains a major challenge because optimal surface characteristics are essential for successful osseointegration.

Titanium surfaces commonly become hydrophobic during shipment, storage, and clinical handling under ambient conditions, leading to diminished biocompatibility [16]. In contrast, surface treatments such as glow discharge [17,18] and ultraviolet (UV) photocatalysis have been shown to restore hydrophilicity by modifying surface properties, including the titanium-to-oxygen ratio, oxide layer thickness, crystallinity, and organic impurity levels [19]. These modifications are associated with enhanced cell adhesion, osteoconductivity, and early-stage osseointegration. In a previous study, we demonstrated that titanium nitride (TiN) coatings applied to commercially pure titanium via arc ion plating maintained cellular compatibility while substantially improving both mechanical and chemical resistance. Additionally, TiN coatings preserve a smooth surface topography over time, potentially reducing the risk of peri-implantitis [20]. However, conventional surface modification techniques often require complex procedures and expensive equipment and may be unsuitable for implants with intricate surface morphologies. UV treatment struggles to achieve uniform and durable hydrophilicity due to limited light penetration and surface coverage.

Ozone is a strong oxidizing agent and is widely used for sterilization, deodorization, and decolorization in various industries [21]. Ozone gas can be dissolved into water by discharge or electrolysis to produce ozonated water, which disperses fine bubbles throughout the medium. Ozonated water exhibits strong sterilizing and cleaning properties and is employed in applications ranging from food sanitation to semiconductor fabrication [22]. In dentistry, ozonated water has emerged as a promising agent for managing peri-implantitis because of its potent antimicrobial activity and minimal toxicity to oral tissues [23].

Ozone nanobubble water (NBW3), created by compressing ozone-containing microbubbles, shares many characteristics with conventional ozonated water [24]. Microbubbles—defined as having diameters less than 50 μm—collapse in the liquid phase, generating free radicals. Nanobubbles, which are typically less than 1 μm in diameter, are formed from microbubbles under elevated temperature and pressure conditions. NBW3 increases the hydroxyl ion concentration at the gas–liquid interface during collapse, thereby suppressing ozone dissolution into the surrounding environment and preserving oxidative potential for several months under UV-protected conditions. NBW3 is also widely recognized as containing submicron-sized bubbles with unique physicochemical properties, in which ozone is retained as nanobubbles with diameters ranging from 50 to 500 nm [25].

In our previous investigation, titanium disks stored in NBW3 maintained higher levels of hydrophilicity and exhibited superior cell compatibility compared to those stored under ambient conditions due to reduced hydrocarbon contamination [26]. During the initial phases of osseointegration, the bone–implant interface is characterized by the formation and remodeling of the extracellular bone matrix, which serves as a critical scaffold for osteoblast adhesion, proliferation, and differentiation. This bone matrix, primarily composed of collagen type I, non-collagenous proteins, and mineral components, plays a central role in establishing mechanical and biochemical interactions with the implant surface [27,28]. Optimizing implant surface conditions to support early matrix deposition and mineralization is therefore crucial for achieving stable bone anchorage. Given the importance of this dynamic interface, preserving the bioactivity of titanium implants through storage in NBW3 may enhance early osseointegration by facilitating favorable interactions between the implant surface and newly formed bone matrix. This in vivo pilot study evaluated NBW3 as a potential implant storage medium for promoting rapid osseointegration.

Based on the objectives of this study, the following null hypotheses were tested:

(1) There is no difference in the degree of early osseointegration between titanium implants stored in NBW3 and those stored under ambient conditions.
(2) Storage in NBW3 does not influence bone-to-implant contact (BIC) or peri-implant bone formation.
(3) The preservation of surface hydrophilicity via NBW3 does not affect the biological performance of titanium implants in vivo.

## 2. Materials and Methods

### 2.1. Ozone Nanobubble Water

Ozone nanobubble water (NBW3; NANODENTALα, KYOCERA Corporation, Kyoto, Japan) was employed as a light pink aqueous solution containing ozone at a concentration of 1.5 mg/L with a pH of 7.5 (Figure S1, Table S1). The manufacturing technology for NBW3 was jointly developed by the REO Research Institute (Miyagi, Japan) and the National Institute of Advanced Industrial Science and Technology (AIST, Tokyo, Japan). The average diameter of the nanobubbles was approximately 140 nm, with a standard deviation of about 30 nm, as reported by dynamic light scattering (DLS) measurements [29].

### 2.2. Titanium Implant Bodies

**Figure 1:**
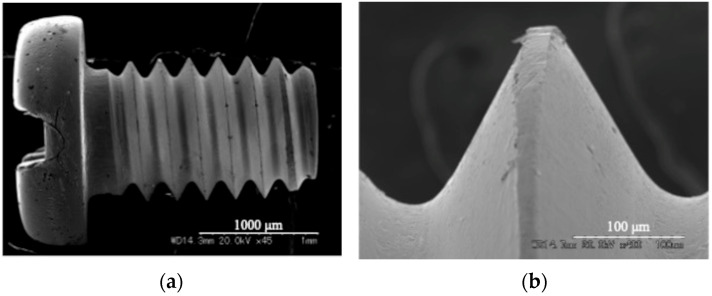
SEM images of the titanium implant’s surface morphology: (**a**) the overall view of the implant (45× magnification); (**b**) an enlarged view of the threaded region (400× magnification).

Screw-shaped titanium implants (JIS Type 2; diameter: 1.0 mm; length: 2.0 mm) were fabricated from commercially pure titanium (TW340; Nishimura Co., Ltd., Fukui, Japan) using micro-header and micro-form rolling processes, cleaned in absolute ethanol using an ultrasonic bath, and sterilized by autoclaving (Figure 1).

All implants were subjected to hydrophilization via glow discharge plasma treatment (PIB-10, Vacuum Device, Ibaraki, Japan) under an argon atmosphere (1.3 × 10^−6^ Pa) for 3 min. Following treatment, the implants were divided into 2 groups: the experimental group, in which the implants were stored in NBW3 for 7 days, and the control group, in which the implants were stored under atmospheric conditions. The 7-day storage period was selected based on our previous study [26], which demonstrated that NBW3 effectively preserved surface hydrophilicity and minimized hydrocarbon contamination for at least one week under UV-shielded conditions. This duration was considered appropriate to simulate realistic short-term clinical storage conditions while maintaining sufficient surface bioactivity prior to implantation. Both mechanical and histological evaluations of osseointegration were conducted.

### 2.3. In Vivo Implantation in Sprague Dawley Rats

Initially, 24 male Sprague Dawley (SD) rats (8 weeks old; Hokudou, Sapporo, Japan) were included in the study. However, data from animals affected by procedural errors or equipment malfunctions were excluded from the final analysis. Animals were housed in a controlled environment (12 h light/dark cycle, constant temperature, and humidity) at the Animal Experiment Center of Hokkaido University of Health Sciences, and water and standard laboratory chow were provided ad libitum. All procedures adhered to institutional animal care guidelines and were approved by the Animal Experiment Committee of Hokkaido University of Health Sciences (Approval No. 093).

**Figure 2:**
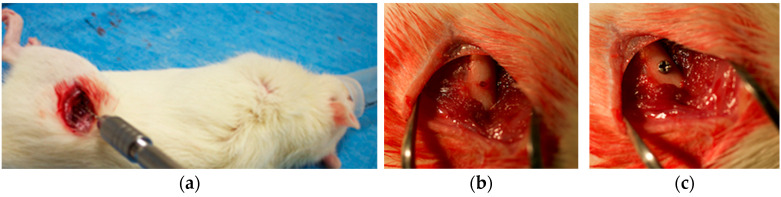
Surgical procedure for implant placement in rat femurs: (**a**,**b**) bone defect created using precision pin vice; (**c**) placement of titanium implants in femurs of rats.

Anesthesia was administered via isoflurane inhalation, followed by local infiltration. A standardized cylindrical bone defect (1 mm diameter, 2 mm depth) was created in the femoral shaft using a precision pin vice (Tsugawa Trading, Tokyo, Japan) (Figure 2a,b). Rats were randomly assigned to the control or experimental group and received one implant in each femur (n = 6 per group per time point) (Figure 2c). Animals were monitored daily for postoperative complications, including >10% body weight loss, signs of infection, wound dehiscence, impaired mobility, and dehydration. No animals met the exclusion criteria. Euthanasia through isoflurane inhalation followed by carbon dioxide administration was performed 14 or 28 days post-surgery. All assessments were performed by a single investigator under standardized conditions to minimize variability.

### 2.4. Mechanical Analysis

#### 2.4.1. Removal Torque Measurement

The removal torque was evaluated using a digital torque gauge (ATG6CN, Tohnichi, Tokyo, Japan) 14 and 28 days post-implantation (n = 6 per group per time point).

#### 2.4.2. Scanning Electron Microscopy (SEM) and Energy-Dispersive X-Ray (EDX) Analysis

Following retrieval, the implant specimens were processed using a critical point dryer (HCP-2, Hitachi, Tokyo, Japan) and sputter-coated using an ion coater (IB-3, EIKO, Tokyo, Japan). Surface morphology and bone-like tissue attachment were assessed by SEM (S-3500, Hitachi, Tokyo, Japan). EDX analysis (S-3500, Hitachi, Tokyo, Japan) was used to quantify the calcium-to-phosphorus (Ca/P) atomic ratios (20 kV; working distance: 15 mm) at the bone–implant interface. No volume or density corrections were applied; relative ratios were compared between groups.

### 2.5. Histological Preparation and Analysis

#### 2.5.1. Sample Processing

After euthanasia, the femurs containing implants were harvested and embedded in polyester resin (Rigolac, Nisshin EM, Tokyo, Japan). Sections perpendicular to the implant axis were obtained using a diamond band saw (Exakt BS 310 CP, Norderstedt, Germany) and subsequently polished.

#### 2.5.2. Soft X-Ray Imaging

Sections approximately 120 μm thick were imaged using a soft X-ray apparatus (SOFRON BST-1505CX, Soken, Tokyo, Japan) with the following settings: a 40 kV tube voltage, a 1 mA tube current, and 1 s of exposure. Grayscale surface plots were analyzed using ImageJ software version 1.51 (NIH, Bethesda, MD, USA).

#### 2.5.3. Histological Staining and Bone-to-Implant Contact (BIC) Analysis

The specimens were further sectioned to approximately 50 μm and stained with basic fuchsin and methylene blue (Wako, Osaka, Japan). Bone–implant contact (BIC) was quantitatively evaluated using ImageJ software (NIH, Bethesda, MD, USA) following microscopic observation (VHX-1000, Keyence, Osaka, Japan) by calculating the length of the interface where the bone was in direct contact with the implant surface.

### 2.6. Statistical Analysis

All quantitative data are presented as the mean ± standard deviation (SD). Prior to analysis, normality was assessed using the Shapiro–Wilk test, followed by Levene’s test to evaluate the homogeneity of variances. For comparisons between two groups, an unpaired Student’s *t*-test was applied when equal variances were assumed; otherwise, Welch’s *t*-test was used. When the assumptions for the parametric tests were not satisfied, the Mann–Whitney U test was employed as a non-parametric alternative. A *p*-value of < 0.05 was considered statistically significant. The choice of statistical tests was based on the distribution and variance characteristics of the data.

The sample size was determined based on previous studies with similar experimental designs and was considered sufficient to detect biologically meaningful differences in osseointegration outcomes [26]. No data were excluded from the analysis, and all statistical evaluations were blindly conducted using SPSS Statistics version 29.0 (IBM Corp., Armonk, NY, USA).

## 3. Results

### 3.1. Removal Torque Analysis

**Figure 3:**
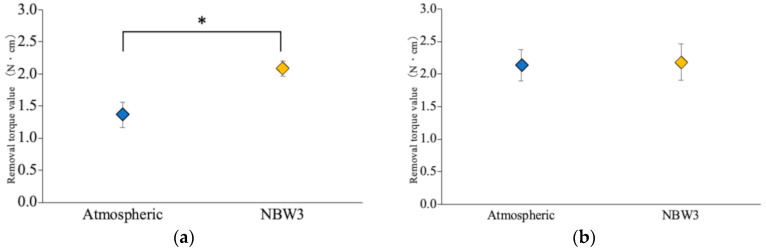
Removal torque values at the time of implant retrieval: (**a**) 14 days post-implantation; (**b**) 28 days post-implantation (n = 6, * *p* < 0.05).

Fourteen days post-implantation, the implants stored under atmospheric conditions exhibited a removal torque of 1.37 ± 0.20 N·cm, whereas those stored in NBW3 exhibited a significantly higher value of 2.08 ± 0.12 N·cm, representing a 1.5-fold increase. By day 28, the torque value increased to 2.13 ± 0.24 N·cm for the atmospheric group and 2.18 ± 0.28 N·cm for the NBW3 group. No statistically significant difference was observed between the groups at this time point, with both groups exceeding 2.00 N cm. Notably, the implants stored in NBW3 achieved the torque threshold by day 14, whereas the atmospheric group required 28 days to reach comparable levels (Figure 3). The results of the statistical analysis are provided in Table S2.

### 3.2. SEM and EDX Observations

**Figure 4:**
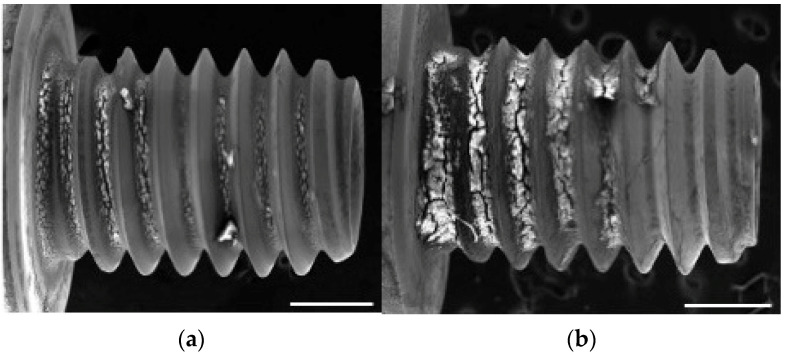

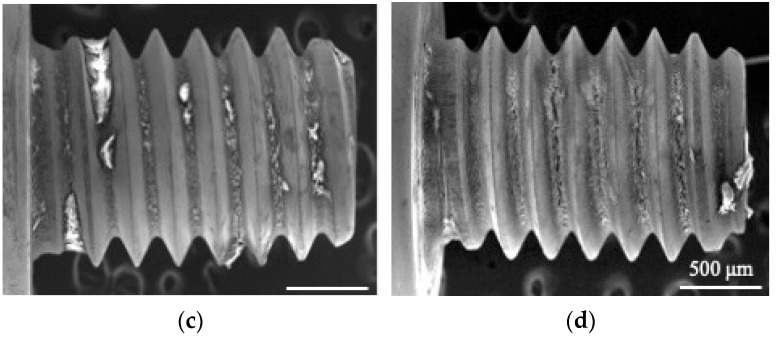
SEM images of retrieved implants (50× magnification): (**a**) 14 days after implantation under atmospheric storage; (**b**) 14 days after implantation with NBW3 storage; (**c**) 28 days after implantation under atmospheric storage; (**d**) 28 days after implantation with NBW3 storage.

**Figure 5:**
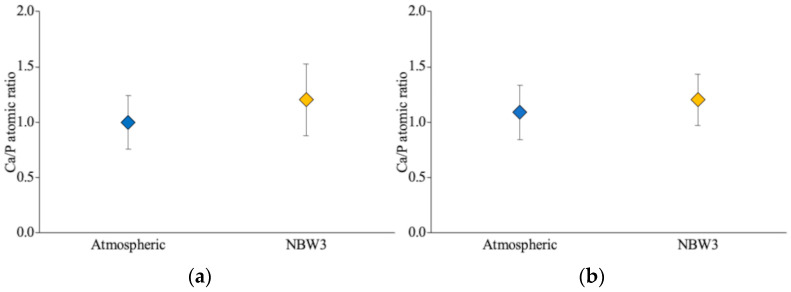
Calcium-to-phosphorus (Ca/P) atomic ratios on implant surfaces: (**a**) 14 days post-implantation; (**b**) 28 days post-implantation (n = 6, *p* < 0.05; no statistically significant differences were observed).

SEM was performed 14 days post-implantation and revealed more extensive attachment of bone-like tissue on the NBW3-stored implants than on those stored under atmospheric conditions (Figure 4a,b). EDX analysis showed that the Ca/P atomic ratio of the attached tissue was 1.0 ± 0.24 in the atmospheric group and 1.20 ± 0.32 in the NBW3 group, with both values exceeding 1.0 (Figure 5a). At 28 days, the SEM images revealed denser tissue accumulation within the valleys of the implant surfaces in both groups compared with the findings at 14 days (Figure 4c,d), with no observable differences in tissue coverage. At this time, the EDX analysis showed a Ca/P ratio of 1.09 ± 0.25 in the atmospheric group, with no significant difference between groups (Figure 5b). The results of the statistical analysis are provided in Supplementary Table S3.

### 3.3. Soft X-Ray Analysis

**Figure 6:**
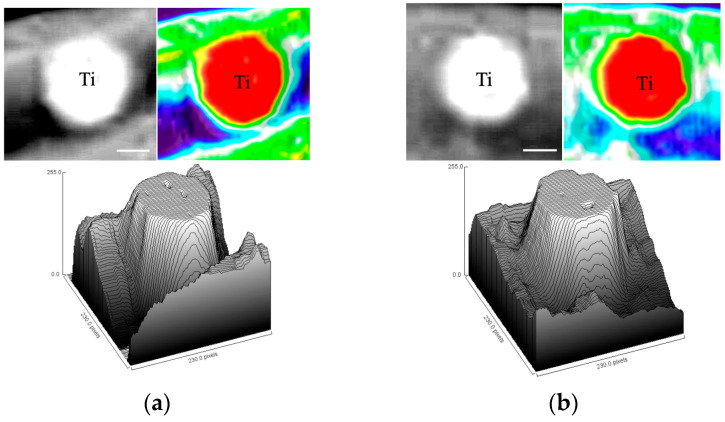

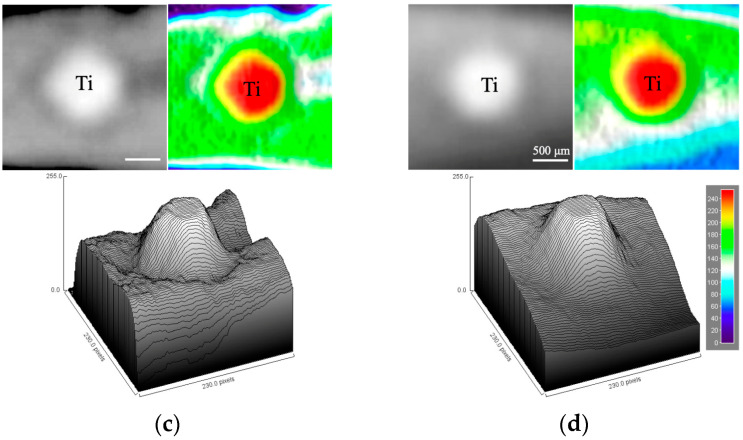
Soft X-ray images of peri-implant bone: (**a**) 14 days post-implantation under atmospheric storage; (**b**) 14 days post-implantation with NBW3 storage; (**c**) 28 days post-implantation under atmospheric storage; (**d**) 28 days post-implantation with NBW3 storage. “Ti” denotes the titanium implant.

Soft X-ray radiography 14 days post-implantation showed indistinct or limited bone formation surrounding the implants in both groups (Figure 6a,b). However, a grayscale surface plot analysis performed using ImageJ revealed greater radiopacity around the NBW3-stored implants, suggesting a trend toward enhanced early bone formation. At 28 days, radiopaque features surrounded the implants in both groups with comparable intensity (Figure 6c,d).

### 3.4. Histological Observation and BIC Ratio

**Figure 7:**
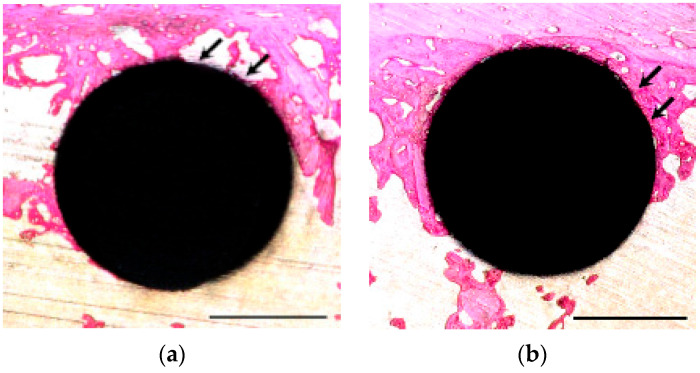

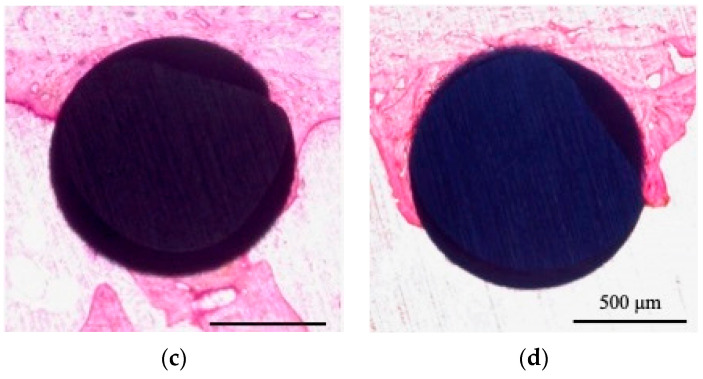
Basic fuchsin–methylene blue double-stained histological images of the peri-implant tissue: (**a**) 14 days post-implantation under atmospheric storage. Unstained areas adjacent to the implant surface indicate the absence of newly formed bone (arrows); (**b**) 14 days post-implantation with NBW3 storage. Newly formed bone was identified as the area stained from red to pink and was observed adjacent to the implant surface (arrows); (**c**) 28 days post-implantation under atmospheric storage; (**d**) 28 days post-implantation with NBW3 storage.

**Figure 8:**
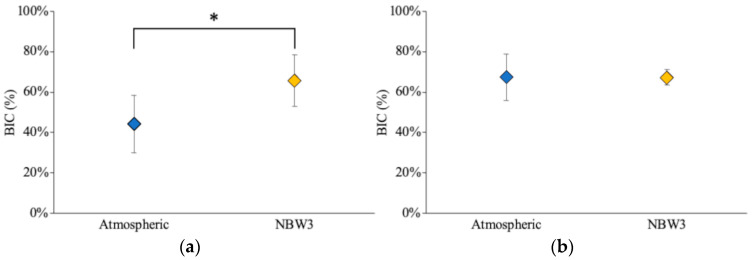
Bone-to-implant contact (BIC) ratios: (**a**) 14 days post-implantation; (**b**) 28 days post-implantation (n = 6, * *p* < 0.05).

Histological staining at 14 days revealed that the NBW3-stored implants exhibited more extensive formation of immature, reddish-purple bone tissue than those stored under atmospheric conditions (Figure 7a). The quantitative analysis showed a significantly higher BIC ratio in the NBW3 group (65.74 ± 12.65%) than in the atmospheric group (44.04 ± 14.25%), corresponding to a 1.4-fold increase (Figure 8a). By 28 days, mature bone formation was evident around the implants in both groups, with no substantial changes in tissue morphology relative to the 14-day results (Figure 7b). The BIC ratio exceeded 65.0% in both groups at 28 days (Figure 8b). Importantly, the NBW3-stored implants reached a BIC value above 60% as early as day 14, whereas the atmospheric implants required 28 days to achieve this level. The results of the statistical analysis are provided in Supplementary Table S4.

## 4. Discussion

In this study, we used the femoral bone of SD rats as the implantation site to assess the potential of NBW3 to promote rapid bone formation and osseointegration. As a pilot study informed by prior in vivo data [26], we anticipated a large effect size; thus, six samples per group were deemed sufficient to achieve statistical power while adhering to ethical standards regarding animal use. Although machined-surface implants are no longer common in clinical settings, they were intentionally selected to establish a baseline osseointegration profile, building upon previous findings demonstrating their reliability [30,31,32].

At 14 days, the NBW3-stored implants showed significantly greater removal torque, bone-to-implant contact (BIC), and Ca/P atomic ratio compared to the controls, indicating enhanced early osseointegration. These differences were not observed at 28 days, suggesting that NBW3 primarily accelerates the initial bone integration phase.

Osseointegration, first defined by Brånemark, refers to the direct structural and functional connection between living bone and the surface of a load-bearing implant without interposing soft tissue [4]. Among the emerging strategies used to enhance this process, a variety of surface modification techniques have been proposed and studied, including ultraviolet (UV) treatment, laser irradiation, plasma treatment, acid etching, sandblasting, and nanostructuring. These approaches aim to enhance biological performance by improving surface topography, chemistry, and hydrophilicity, thereby facilitating cell adhesion, proliferation, and differentiation. For example, UV-C photofunctionalization has been shown to increase titanium surface hydrophilicity and reduce organic contamination, significantly improving early-stage osteoblast activity and bone–implant contact [33]. Similarly, nanostructured surfaces mimic the extracellular matrix, promoting the osteogenic differentiation of mesenchymal stem cells [34]. Acid-etched and sandblasted (SLA) surfaces increase surface roughness, enhancing mechanical interlocking and osseointegration [10]. Among these, laser-based techniques have shown considerable promise. In particular, Er:YAG laser irradiation—representing a form of non-contact, photothermal surface conditioning—has been reported to reduce microbial load and significantly enhance surface wettability, thereby creating a favorable environment for bone integration and healing [35,36,37]. However, many of these approaches require specialized equipment, may be cost-prohibitive, and often necessitate immediate clinical use post-treatment due to rapid re-contamination or hydrophobic recovery. In contrast, NBW3 offers a unique advantage as a post-manufacturing storage solution that preserves the bioactive surface state without altering the topography or requiring re-treatment immediately before implantation.

In addition to topographical and chemical modifications, the biocompatibility of the implant surface—defined as the ability to perform with an appropriate host response—is a critical factor influencing early and long-term osseointegration [38,39]. Surface treatments that improve wettability and reduce contamination are known to enhance biocompatibility by supporting protein adsorption, cellular adhesion, and osteogenic differentiation. Achieving rapid and robust osseointegration is critical for implant success, especially under early or immediate loading conditions. In our previous study [26], we demonstrated that NBW3 storage effectively preserves the hydrophilicity of titanium surfaces by preventing hydrocarbon contamination. XPS analysis showed that the C1s peak intensity remained low in the NBW3-stored titanium disks, similar to the levels observed immediately after glow discharge, even after 28 days of storage. In contrast, disks stored in air or acetone showed a significant increase in hydrocarbon-related peaks. Additionally, contact angle measurements revealed that the NBW3-stored samples maintained the lowest values over time, indicating the long-term stability of surface wettability.

Blood and tissue fluids function as colloidal solutions enriched with extracellular matrix proteins. Titanium surfaces contaminated with hydrocarbons become hydrophobic, resulting in a lower surface energy, which interferes with protein functionality and impedes cell adhesion, likely due to microbubble formation [40]. Conversely, hydrophilic surfaces facilitate protein adsorption and cellular attachment, which are critical for effective wound healing and osseointegration [41,42]. In a previous study, we showed that NBW3 storage preserves the hydrophilic nature of titanium surfaces by preventing hydrocarbon accumulation [26]. Similar findings were reported by Takahashi et al., who found that NBW3 maintained high surface wettability equivalent to that achieved through UV-C treatment [25].

The initiation of osseointegration involves the deposition of bone matrix proteins at the implant–bone interface, including an amorphous layer enriched with osteocalcin and osteopontin produced by immature osteoblasts [43]. Osteocalcin binds hydroxyapatite and calcium ions, facilitating osteoblast recruitment, whereas osteopontin enhances bone bonding via integrin interactions. These events coincide with increased alkaline phosphatase (ALP) activity, a marker of early osteogenesis [44]. Notably, low ozone concentrations (0.3–0.8 ppm) induce mild oxidative stress, which activates the Nrf2 pathway and helps reduce inflammation [45]. Our previous study confirmed that low-dose NBW3 enhances the proliferation, adhesion, and ALP activity of human mesenchymal stem cells (hMSCs) [26], suggesting that NBW3 promotes early-stage bone healing through intracellular pathway activation.

After implantation, biological events similar to fracture healing restore the mechanical load-bearing capacity [46,47]. The soft callus begins with endochondral ossification and is accompanied by periosteal thickening and new bone formation beneath the periosteum. Peak callus formation typically occurs around 14 days post-implantation.

In the current study, the implants stored in NBW3 exhibited a 1.5-fold increase in removal torque and a 1.4-fold increase in the bone–implant contact (BIC) ratio compared to the atmosphere-stored implants at 14 days. By 28 days, these values were similar between the groups, indicating that NBW3 primarily accelerates early healing rather than altering the outcome. The SEM findings support this, showing enhanced bone-like tissue formation in the NBW3-stored implants. Additionally, an EDX analysis revealed a higher Ca/P atomic ratio (1.20 ± 0.32) in the NBW3-stored samples versus 1.0 ± 0.24 in the controls. As physiological bone typically presents with a Ca/P ratio ranging from 1.0 to 1.66 [48,49,50,51], these findings show that NBW3 promotes early mineralization within the normal range.

By 28 days, removal torque, BIC, and SEM assessments were comparable between the groups, reinforcing the notion that NBW3 primarily enhances early osseointegration kinetics without compromising long-term integration quality. Understanding these time-dependent differences may provide insights into the molecular events underlying implant healing.

Although various surface treatments exist to improve implant integration [52], most implants are stored in atmospheric conditions for extended periods before use. Early healing failure—most commonly occurring within 2–4 weeks—has been linked to the transition from primary to secondary stability [53]. Thus, enhancing early bone formation is critical to ensure timely secondary fixation and reduce the risk of failure. In this context, NBW3 storage is a simple and cost-effective strategy for maintaining surface bioactivity and supporting early or immediate loading protocols.

To our knowledge, this is the first in vivo study to demonstrate that NBW3 storage prevents titanium surface aging and accelerates osseointegration. NBW3 has several advantages: it requires no specialized equipment, is compatible with screw-shaped implants, and preserves surface bifunctionality. Taken together, our findings suggest that NBW3 enhances early bone formation, as evidenced by the mechanical and histological metrics observed at 14 days.

Accordingly, the null hypotheses of this study—namely that (1) NBW3 storage does not improve early osseointegration, (2) NBW3 storage has no effect on bone-to-implant contact, and (3) surface hydrophilicity preservation does not influence biological performance—were all rejected.

Despite the promising results, this study has several important limitations that must be considered when interpreting the findings. First, the use of a small animal model (Sprague Dawley rats) limits the direct translatability of the results to human clinical scenarios. Rats possess faster bone metabolism and a smaller bone volume compared to humans, meaning the speed and extent of osseointegration may be overestimated. Therefore, the effects of NBW3 observed in this model may not fully replicate the effects in larger animals or humans with more complex bone healing environments. Second, the study was conducted under highly controlled laboratory conditions, including standardized surgical procedures, implant placement, and post-operative care. While this approach ensures experimental consistency, it may not accurately reflect the variability encountered in clinical practice, such as patient-related factors (e.g., systemic disease, smoking, and medications), bone quality, and different anatomical sites. Third, the follow-up periods were relatively short (14 and 28 days), focusing on early osseointegration phases. Although early mechanical stability and histological integration are critical indicators, the long-term behavior of NBW3-treated implants—such as bone remodeling dynamics, late-stage mineralization, and resistance to peri-implant inflammation—remains unexplored. Thus, the longevity and durability of the observed benefits are not yet fully understood. Fourth, this study only employed one implant surface type (machined titanium) to establish a baseline response. While this enhances the internal validity, it limits generalizability across the diverse surface treatments used in modern implantology (e.g., SLA, anodized, and HA-coated surface treatments). It is unclear whether NBW3 would yield comparable effects on roughened or chemically modified surfaces that already possess high bioactivity. Fifth, although the null hypotheses were statistically rejected based on significant intergroup differences, the pilot nature of the study with a small sample size per group (n = 6) limits the statistical power and generalizability of the results. Larger sample sizes with stratified controls would enhance statistical robustness and reduce variability in future investigations. Finally, the oxidative potential of NBW3, which underpins its bioactivity, may be affected by storage conditions such as light exposure, temperature, and time. These parameters were not exhaustively characterized or controlled in the present study. For broader clinical application, standardized protocols for NBW3 production, storage, and shelf-life management must be established to ensure reproducibility and safety.

## 5. Conclusions

This study demonstrated that storing titanium dental implants in ozone nanobubble water (NBW3) effectively preserved surface hydrophilicity and enhanced early-stage osseointegration. Implants stored in NBW3 exhibited significantly higher removal torque values and bone-to-implant contact (BIC) ratios 14 days post-implantation compared to those stored under atmospheric conditions. These findings were supported by SEM and EDX analyses, which revealed greater bone-like tissue attachment and increased mineralization around the NBW3-stored implants. Although no significant differences were observed at 28 days, the accelerated early healing observed in the NBW3 group suggests that this storage method may contribute to improved initial implant stability, a critical factor for early or immediate loading. NBW3 represents a simple, non-invasive, and clinically feasible approach to mitigating the effects of titanium surface aging and enhancing implant bioactivity. Future studies involving larger animal models with longer follow-up periods are warranted to validate these findings and assess their long-term clinical relevance.

## Data Availability

The original contributions presented in this study are included in the article/Supplementary Materials. Further inquiries can be directed to the corresponding author.

## Supplementary Materials

The following supporting information can be downloaded at https://www.mdpi.com/article/10.3390/ma18133156/s1, Figure S1: Appearance of NBW3 used in this study; Table S1: Physicochemical properties and elemental composition of NBW3; Table S2: Summary of statistical tests for removal torque measurement; Table S3: Summary of statistical tests for calcium-to-phosphorus (Ca/P) atomic ratios; Table S4: Summary of statistical tests for BIC ratio. Reference [54] is cited in the Supplementary Materials.
